# Schools, Air Pollution, and Active Transportation: An Exploratory Spatial Analysis of Calgary, Canada

**DOI:** 10.3390/ijerph14080834

**Published:** 2017-07-25

**Authors:** Stefania Bertazzon, Rizwan Shahid

**Affiliations:** 1 Department of Geography, University of Calgary, University of Calgary, Calgary, AB T2N 1N4, Canada; bertazzs@ucalgary.ca; 2 Alberta Health Services, Calgary, AB T2W 3N2, Canada

**Keywords:** spatial analysis, air pollution, active transportation, walkability, schools, children, proximity, public health, GIS (geographic information systems), Calgary

## Abstract

An exploratory spatial analysis investigates the location of schools in Calgary (Canada) in relation to air pollution and active transportation options. Air pollution exhibits marked spatial variation throughout the city, along with distinct spatial patterns in summer and winter; however, all school locations lie within low to moderate pollution levels. Conversely, the study shows that almost half of the schools lie in low walkability locations; likewise, transitability is low for 60% of schools, and only bikability is widespread, with 93% of schools in very bikable locations. School locations are subsequently categorized by pollution exposure and active transportation options. This analysis identifies and maps schools according to two levels of concern: schools in car-dependent locations and relatively high pollution; and schools in locations conducive of active transportation, yet exposed to relatively high pollution. The findings can be mapped and effectively communicated to the public, health practitioners, and school boards. The study contributes with an explicitly spatial approach to the intra-urban public health literature. Developed for a moderately polluted city, the methods can be extended to more severely polluted environments, to assist in developing spatial public health policies to improve respiratory outcomes, neurodevelopment, and metabolic and attention disorders in school-aged children.

## 1. Introduction

The association between air pollution and adverse health effects is well documented in the literature [1,2,3]. Air pollution exposure at early ages may have even more serious effects, triggering the onset of environmental allergies [4,5]. A person can be exposed to air pollution while in their home, at work, while commuting to work, while exercising or recreating, and in a variety of other situations. Residential location, lifestyle, and socioeconomic status are only some of the factors that contribute to an individual’s exposure. Complex interactions across a large variety of factors occur mostly over space, within and beyond cities. Spatial analysis therefore is not simply a tool: it is an approach to public health research. Known as spatial thinking in the social and health sciences [6,7], this approach has come to the forefront in several disciplines, as it can cast new light on location and spatial interactions. Spatial thinking in the health and social sciences encompasses various forms of geographical and spatial research, including geographical information science and spatial analysis, which rely on spatial data [8]. Spatial data exhibit a number of properties that impact quantitative analyses in various ways, requiring the use of specific analytical tools [9]. One of the most serious concerns with spatial data is uncertainty, which is often intrinsic to the data; it is induced by other properties (spatial dependence and heterogeneity) and can be propagated through analytical models [10,11]. While there exists specific analytical tools to deal with uncertainties, perhaps the most successful approach remains a careful exploratory analysis of the data, which only can lead to better model specification.

Air pollution exhibits spatial patterns that are driven by numerous and often interacting factors, including localized emissions (mostly industry and traffic), atmospheric circulation (especially wind), and topography (elevation), among others. The spatial pattern of air pollution, that of exposure, and their association have received proportionally less attention in the literature [12]. This relative lack of research is in part related to the scarcity of information on the spatial patterns of air pollution. While air quality is typically measured frequently and regularly over time, spatial measurements tend to be sparse and irregular, making it hard to understand the variation in air pollution levels at the urban and intra-urban level [13]. Air quality in the proximity of schools is important, as students (as well as parents, teachers, and school staff) spend time in the area, often performing aerobic activities. The severity of air pollution near schools can be aggravated by the presence of school buses, which not only drive to and from school at least twice daily, but also often idle for extended periods while students come in and out of school. These vehicles, often run on diesel fuel, emit high levels of combustion gases and particulate matter through exhaust pipes that are just at the height of the respiratory organs of young children [14]. Children attending schools in highly polluted areas may not only be at risk of developing respiratory problems but also at risk of high BMI [4], which may in turn increase a number of health risks. Studies also show that traffic related air pollution may have harmful effects on neurodevelopment [5], and be linked with metabolic disorders [4] and fluctuation in children’s attention [15].

Air pollution in urban settings is mainly associated with industrial activities and transportation. In order to reduce pollution associated with commuting and other daily activities, active forms of transportation have been proposed and are generally encouraged. Active forms of transportation include typically walking, biking, and using public transit [16]. Active transportation is also encouraged as a way of achieving recommended daily physical activity levels, which can reduce health risks and obesity [17], the root cause of many health problems. Nonetheless, active transportation is not always a feasible alternative to the use of private automobile, primarily because of distance, i.e., destinations can be far away, and because of the characteristics of the built environment, i.e., urban environments may or may not be safe and conducive of walking [18]. In order to assess the feasibility of daily walking, the concept of walkability has emerged in the literature. While neighbourhood walkability does not determine active transportation, studies have shown that people living in highly walkable neighbourhoods take more steps per day and walk more for transport than those living in less walkable neighbourhoods [19,20]. In children, high walkability is positively associated with active park use [21,22] and overall higher levels of physical activity [23].

Walkability is generally assessed by the distance to major services and amenities in the neighbourhoods [24,25,26]. It can also be determined by a number of built environment characteristics [18], such as sidewalk availability, connectivity, and width; road width, signal availability, land use, intersection density, lighting, trees, sidewalk continuity, road connectivity, traffic volume, security and safety, among others [26,27]. Over the past two decades, several walkability indices have been developed [24,25,28,29,30,31,32,33,34]. The absence of a standardized method with a consistent set of variables makes it cumbersome to calculate or replicate indices for comparability across studies. For these reasons, the Walkscore™ (REDFIN, Seattle, WA, USA) index [35] has gained popularity in recent years. The walk score of a location is obtained as a weighted function of the distance of local amenities combined with a distance decay function [35,36].

High active transportation scores are not synonymous with active communities; however, walkability refers to built environments that promote walking. As such, walkability is generally considered beneficial, both from a health and from an environmental perspective. However, the health benefits of walking, as well as of other forms of active transportation, can be reduced, or even hampered, if active transportation occurs in areas with severe air pollution. Indeed, walkability measures typically disregard air quality. Yet, by engaging in walking and active transportation, we can contribute to the reduction of noxious pollutant emissions, but we may also increase our own exposure to those emissions. Therefore, while the environmental benefits of active transportation remain, the health benefits of physical activity can be offset by pollution exposure. As pollution levels vary *spatially* within a city, the health benefits of active transportation depend largely on *where* we engage in such activity. Further, the health benefits of walking to school and playing in the schoolyard depend largely on *where* schools are located with respect to air pollution.

In this study, we present an exploratory spatial analysis of school location in Calgary, Canada. We investigate the location of primary and secondary schools (kindergarten to grade 12) with respect to the residential pattern of school-aged children, active transportation options, and seasonal air pollution patterns. By analyzing active transportation scores of schools, the research identifies transportation options available to students. Locations of concern are identified, where active transportation options are poor, and air pollution levels are relatively high. Further analysis considers air pollution levels in locations where active transportation options are available. This analysis yields an additional set of locations of concern, where active transportation options are available, yet pollution levels are high. This combination may increase exposure levels of children who actively commute to their school.

## 2. Materials and Methods

### 2.1. Study Area

**Figure 1:**
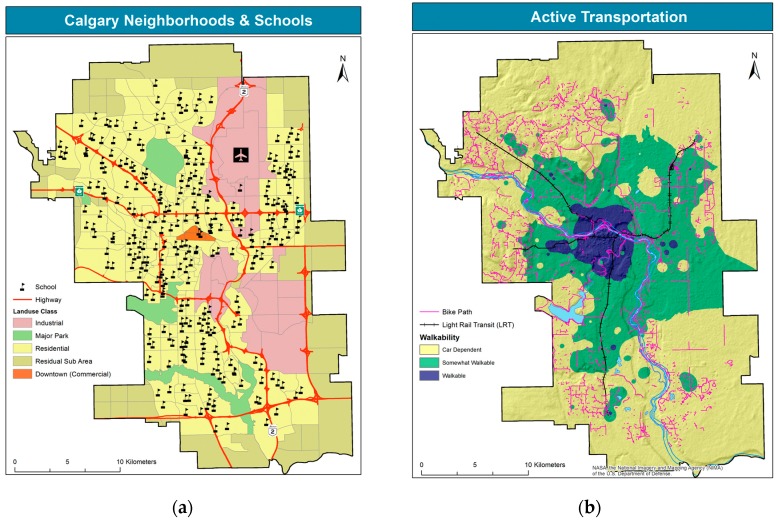
Calgary neighborhoods by land-use class and school location (**a**); and active transportation modes in Calgary (**b**).

Calgary is the fourth largest metropolitan area in Canada, home to over 1.2 million people. Located on the eastern foothills of the Rocky Mountains, at an average elevation of 1000 m, with a 300 m range, it stretches over more than 800 km^2^. Like many north-American cities, Calgary exhibits a segregated land use, with residential areas located in the west, and the industrial park (mostly light manufacturing) in the east. A major international airport is also located in the northeast, and the central business district in the downtown core, as shown in Figure 1a. With prevailing westerly and northerly winds, and west-east degrading slopes, the eastern location of the most polluting facilities provides some protection from pollution to urban residents. However, major transportation corridors, i.e., roads and railway, tend to run in river valley bottoms, constituting a major source of localized pollution. At latitude of about 50 degrees, Calgary experiences substantial seasonal variations. Summers are warm and mild, with average temperatures in the low- to mid-20 degrees Celsius, whereas winters are long and cold, with average temperatures ranging between −20 and −10 degrees Celsius. In the winter months, air pollution levels increase, owing to residential heating and more intense local driving. Overall, Calgary’s air quality is generally considered safe, yet pollution levels exhibit large spatial variation: highest pollution levels are recorded in the east, the downtown core, and along the river valleys and transportation corridors [37,38,39].

Located in the oil rich province of Alberta, Calgary’s economy is linked to the tertiary sector related to oil and gas extraction. With a relatively young and wealthy population, Calgary has developed over the last century as a car-dependent city (Figure 1b), with low population density and sprawling suburbs [40,41]. The current residential pattern reflects the city’s historic radial development, with older dwellings in the city core, and dwelling age decreasing as distance from the city core increases. Demographics follow a similar pattern, with younger families and children located mostly in the outskirts, where walkability is lower, along with accessibility of other urban services. Due to rapid economic and demographic expansion in the last portion of the 20th century, the city followed a sprawling development, with low density, a majority of single-family dwellings, and a heavily car-dependent lifestyle. The construction of schools tends to happen chronologically after the development of new communities, whereas schools tend to remain in inner city areas, which where once occupied by young families. This locational gap, along with families’ choice with respect to specialty and chartered schools, results in long commutes, partly provided by the public school bus system, and partly provided by private vehicles and public transit.

According to current policies of the Calgary board of education (CBE), school bus services are only provided to children whose residence is located at a distance from the school they attend greater than 1.6 km for elementary school students, and 2.4 km for students in the junior high age group [42]. Each school usually has a park and playground that provide students with opportunities for outdoor physical activity. Generally, these parks and playgrounds are open to the public and accessible year-round, providing additional recreational opportunities to all children of the local community [40].

### 2.2. Data

Data were obtained from a number of sources. School locations were obtained from the City of Calgary’s Open Data [43] and were geocoded using ArcGIS 10.5 [44]. Only one school was selected for each dissemination area (DA), to reduce redundancy in air pollution, walkabaility, and other active transportation scores. Following this initial query, spatial analyses presented in this study do not consider school density within each DA. A dissemination area is defined by Statistics Canada as a “small area composed of one or more neighbouring dissemination blocks, with a population of 400 to 700 persons” [45]. At the 2011 census, there were 1436 dissemination areas in Calgary. Dissemination areas are the smallest areas for which socioeconomic and demographic data are collected and released.

Walkability was estimated using Walkscore™ [35], a free web tool that calculates an index of walkability for a given address by using distance of local amenities within one mile (1.6 km) and a distance decay function. Similarly, bikescore and transitscore were calculated for each address. These three scores (walk, bike, transit) were calculated for each primary school in the database. Walkscore values range from 0 to 100 and, based on these values, locations are classified into 3 categories: car-dependent (0–49); low walkability (or somewhat walkable) (50–69) and high walkability (70–100). Bikescore is calculated by the availability of bike infrastructure, bike convenience, and if errands can be accomplished by bike. It can be classified into 3 categories: somewhat bikeable (0–49), bikeable (50–69), and very bikeable (70–100). Walkscore^TM^ also provides a transit score index (0–100) for any given address. Transitscore is calculated by the availability of public transit stops and routes and can be classified into 3 categories: some transit (0–49), good transit (50–69), and excellent transit (70–100).

The regulatory air quality monitoring network in Calgary is managed by the Calgary Region Airshed Zone (CRAZ) and consists of three continuous and eight passive stations, located throughout the city [46]. Continuous stations provide hourly pollution data, whereas passive stations provide monthly records, for a range of pollutants. This network provides a sparse sample of pollution records and a poor representation of the spatial pattern of air pollution over the 800 km^2^ urban area. High-resolution estimates of air pollution over the city were obtained from land use regression (LUR) models [37,38,39] computed by our group based on a large array of spatial data, collected from a network of 50 monitors deployed over two-week campaigns in summer 2010 and winter 2011. LUR estimates for nitrogen dioxide (NO_2_) and fine particulate matter (PM_2.5_) were used in this study.

### 2.3. Methods

The spatial analysis conducted in this study is mostly descriptive and exploratory. It uses standard GIS methods, including mapping and overlay [47]. Further, correlation analysis is conducted using Pearson’s correlation coefficient. A linear correlation between two variables, the Pearson’s correlation coefficient ranges from −1, for perfect negative correlation, to +1 for perfect positive correlation, with 0 indicating independence, or lack of correlation. Under the assumption of normally distributed variables, inferential procedures are used to test the statistical significance of the correlation [48].

Air quality in Canadian cities is evaluated using the air quality health index (AQHI) [49]. AQHI is a composite index based on recorded concentrations of nitrogen dioxide (NO_2_), particulate matter (PM_2.5_), and ozone (O_3_), calculated according to Equation (1).

(1) 100010.4 ×[ (e0.000537×OO3−1)+(e0.000871×NO2−1)+(e0.000478×PM2.5−1)]

It yields values on a scale from 1 to 10+, where values 1–3 indicate low risk, 4–6 moderate risk, 7–10 high risk, and >10 very high risk. The AQHI is issued hourly and posted on Environment Canada website. AQHI is calculated only for the three continuous stations of the air quality regulatory network.

Using spatially detailed air quality estimates in Equation (1), we calculated a pseudo-AQHI (p-AQHI) for each dissemination area where one or more schools are located. LUR models [37,39] provided estimates for nitrogen dioxide (NO_2_) and fine particulate matter (PM_2.5_). NO_2_ LUR models used geographically weighted methods, yielding R^2^ measures of 0.86 for the summer, and 0.73 for the winter. Using standard regression methods, PM_2.5_ LUR models yielded R^2^ measures of 0.75 for the summer, and 0.51 for the winter. In all models, significant predictors were indicators of industrial activity and traffic. In addition, NO_2_ models included wind speed and direction, and population density in the winter [37,39]. Ozone (O_3_) concentration estimates were not available from the LUR study [37,38,39]. Based on the assumption that O_3_ is known to be a regional pollutant, i.e., one that exhibits relatively low spatial variation [50], we used O_3_ data from the regulatory passive stations over the corresponding periods. In order to achieve the highest spatial resolution in the O_3_ records, we computed Thiessen polygons [51] around each of the regulatory stations. With these data, we calculated a summer and a winter p-AQHI index at the dissemination area level. These indices are similar to the AQHI, but they are not directly comparable. Both indices use the same formula; however, AQHI uses recorded pollution concentrations, whereas p-AQHI uses high-resolution spatial estimates of concentrations. Further, AQHI uses pollution data over 3 h [49], whereas p-AQHI is based on 2-week pollution concentrations. p-AQHI values were divided into terciles, corresponding to low, medium, and high pollution values.

## 3. Results

**Figure 2:**
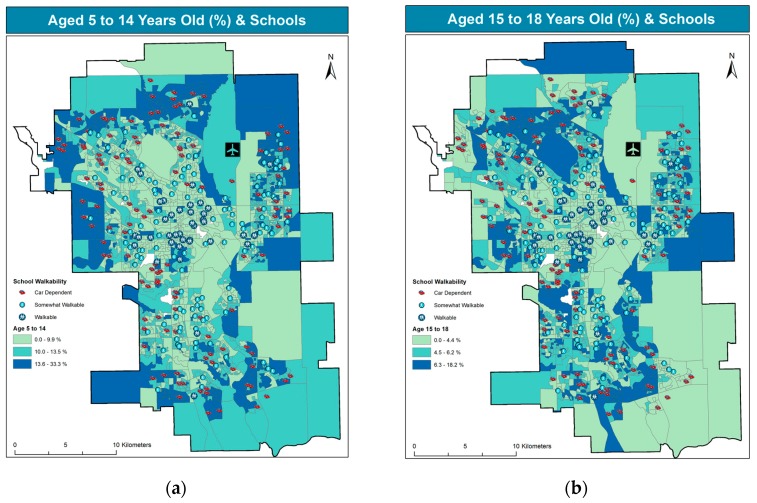
Location of school by walkability and children 5–14 (**a**); and 15–18 (**b**).

Figure 2a presents the residential pattern of children in elementary-to-junior high school age, i.e., grades K to 9, or age 5 to 14. Likewise, Figure 2b shows the residential patterns of senior-high age youth, that is grades 10 to 12, or ages 15 to 18. For each dissemination area where at least one school is located, the walkability score is overlaid. School location is represented by the walkability score, as no explicit school symbols were added to the maps.

There are 279 schools, as shown in Figure 2, with several dissemination areas featuring more than one school The 279 schools are spread across 1436 dissemination areas. A single dissemination area may contain up to 7 schools. While schools are located throughout the city in almost every residential neighborhood, the highest concentration of child population is observed in the fringes of the city, mostly in newly developed neighborhoods. Conversely, school density is higher in inner city areas, which were once home to large numbers of children. Figure 2 further shows that children’s residence and walkability exhibit almost opposite patterns, as the frequency of children is higher in newer, more peripheral communities, where car dependence is highest.

**Figure 3:**
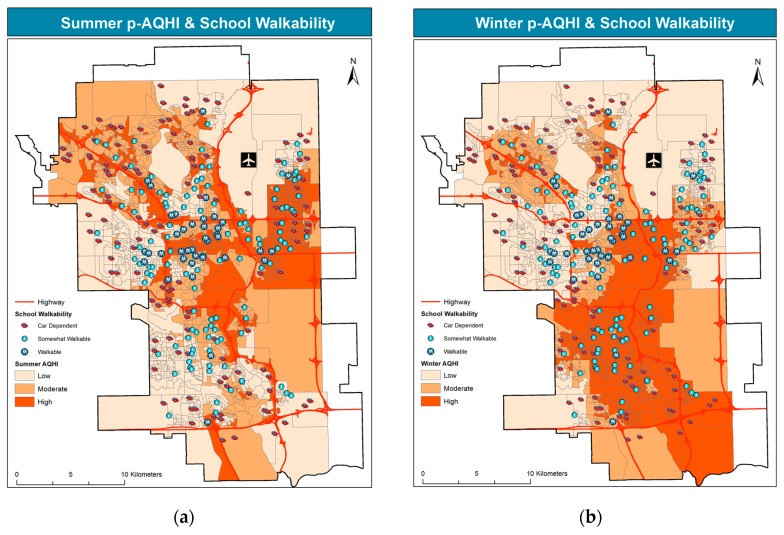
School locations by walkability over summer p-AQHI (**a**) and winter p-AQHI (**b**).

Estimated pollution levels, represented by summer and winter p-AQHI, along with location of schools by walkability, are mapped in Figure 3.

The figure shows that pollution levels tend to be higher along the North-South axis, where the main traffic corridor runs, within a major river valley. Pollution levels are also elevated in the eastern part of the city, in proximity of the main industrial area and the international airport, and strengthened by the prevailing westerly winds. A large number of schools are located in residential areas to the west of the N-S corridor, yet school location tends to coincide with more polluted areas, particularly in the southern part. In the North-West, schools are more frequent along a major road (Crowchild Trail/Highway 1A, shown in Figure 1) where pollution levels are higher. High frequency of schools is also observed in the highly populated and polluted northeast, i.e., east of the airport and close to the industrial area. Summer pollution levels display a more distinct pattern around traffic corridors and industrial areas. Conversely, winter pollution exhibits a more diffused pattern, as residential heating and local driving during the cold season are significant pollution sources in the winter [37]. Whereas schools are attended by children and school staff regularly in the winter season, their outdoors playgrounds remain important destinations for children to play also in the summer, particularly in the most walkable communities.

**Figure 4:**
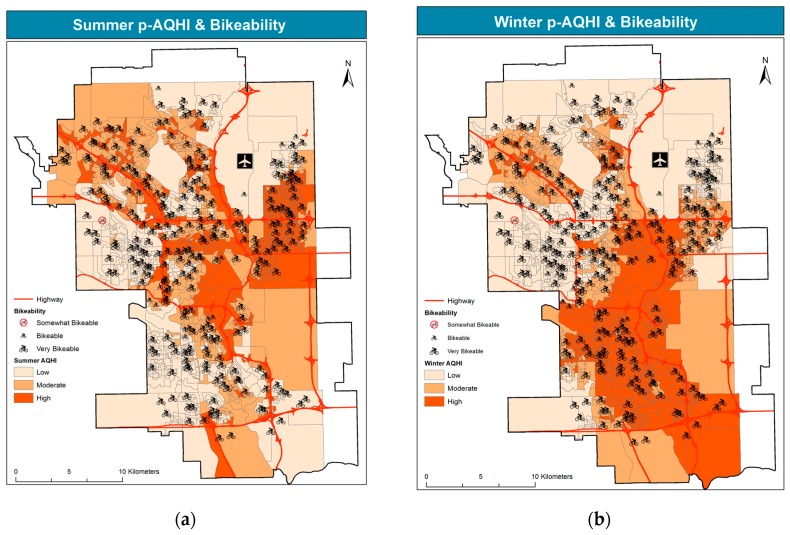
School locations by bikability over summer p-AQHI (**a**) and winter p-AQHI (**b**).

Similar to Figure 3 and Figure 4 overlays school bikability scores to estimated air pollution levels in the summer and winter.

Practically all the schools are in locations that are bikable or very bikable, with minor exceptions in the west/northwest portion of the city. Indeed, most schools score “very bikable”, consistently with the city’s extensive bicycle pathway network, shown in Figure 1. Notably, a large portion of very bikable schools lies in the most polluted parts of the city, particularly the NE in the summer, and a large part of the south in the winter.

**Figure 5:**
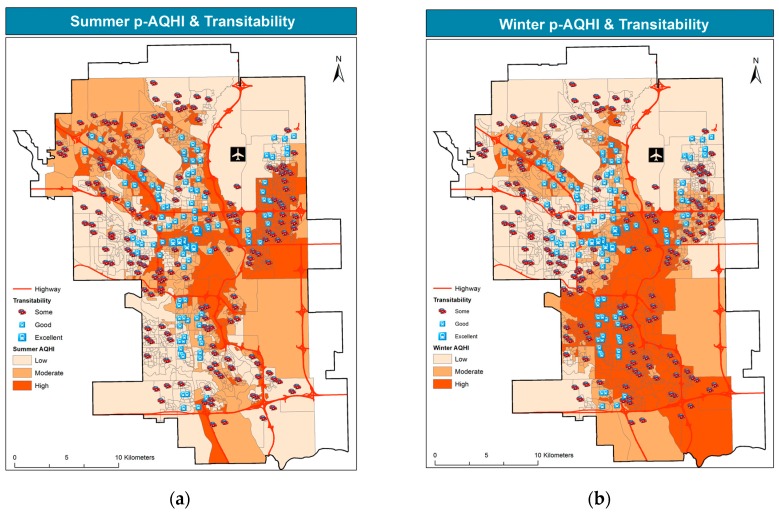
School locations by transitability over summer p-AQHI (**a**) and winter p-AQHI (**b**).

Likewise, Figure 5 shows school location by transit scores, over air pollution levels in summer and winter.

The spatial pattern of transitability shown in the figure resembles a three-prong fork pointing upwards, or north. This pattern reflects the city light rail transit system (LRT). The maps also show that good to excellent transit scores cover only a very small portion of the city, along the main traffic corridors. With few exceptions in the northeastern southern portions, most transitable locations lie in low pollution areas.

Table 1 presents the correlation across air pollution in both seasons, child and total population, and the three active transportation indices.

Several correlations are statistically significant. Relatively high correlation values are only observed within variables of the same category (e.g., across children of different ages and total population). Conversely, all the correlations across variables of different categories are lower than 0.4 in absolute value. The highest correlations are negative, in the 0.3–0.4 range, and are observed for children (both age groups) with walkscore and transitscore. These values indicate that most of the population resides in the lowest walkable and transitable neighbourhoods, and that the negative association between residence and active transportation options is more severe for the child population. Consistent with the widespread pattern shown in Figure 4, the bikability index exhibits low, yet statistically significant correlations. Negative correlations in the 0.2–0.3 range are observed for children in both age groups and AQHI in both seasons. Once again, slightly lower correlation with the same sign is observed for the total population. These correlations suggest that most population resides in less polluted areas, and this correlation is higher for the child population. Of the three active transportation indices, only transit is positively correlated (0.20) with the summer AQHI. This correlation is consistent with the pattern of transitability presented in Figure 5, whereby the more transitable locations lie near the major traffic corridors.

Table 2 summarizes the percentage of schools located in areas with varying levels of pollution (summer and winter) and active transportation modes (walkability, bikability, transitability).

Each of the three panes in the table compares one active transportation index with summer and winter pseudo-AQHI, for each school location. The column totals (identical in both seasons) indicate that almost half of the schools (45%) lie in non-walkable, or car dependent locations; of the remaining half, 41% lie in somewhat-walkable locations, and only 14% in highly walkable locations. Conversely, all the schools lie in bikable locations, with no less than 93% in highly bikable locations. With respect to transit, more than half of the schools (61%) lie in poorly transitable locations, with only 3% in locations with excellent transportability.

The row totals of Table 2, identical in each pane but different in the two seasons, show that approximately 40% of schools lie in low-pollution locations, approximately 30% in medium-pollution locations, and approximately 30% in high-pollution locations. The upper-right cells of each pane represent the most fortunate school locations, where active transportation scores are highest, and air pollution is lowest; whereas the least fortunate locations are those in the lower left corner of each pane, where air pollution is highest and active transportation scores are lowest. While the latter locations are far from ideal, less obvious health risks may be associated with school locations situated in the lower right corner of each pane of Table 2, where active transportation options are available, yet air pollution is high. This percentage of schools (bolded in the table) amounts to 16% in the summer and 20% in the winter with respect to walkability, 25% in the summer and 32% in the winter with respect to bikability, and 10% in the summer and 13% in the winter with respect to transitability.

**Figure 6:**
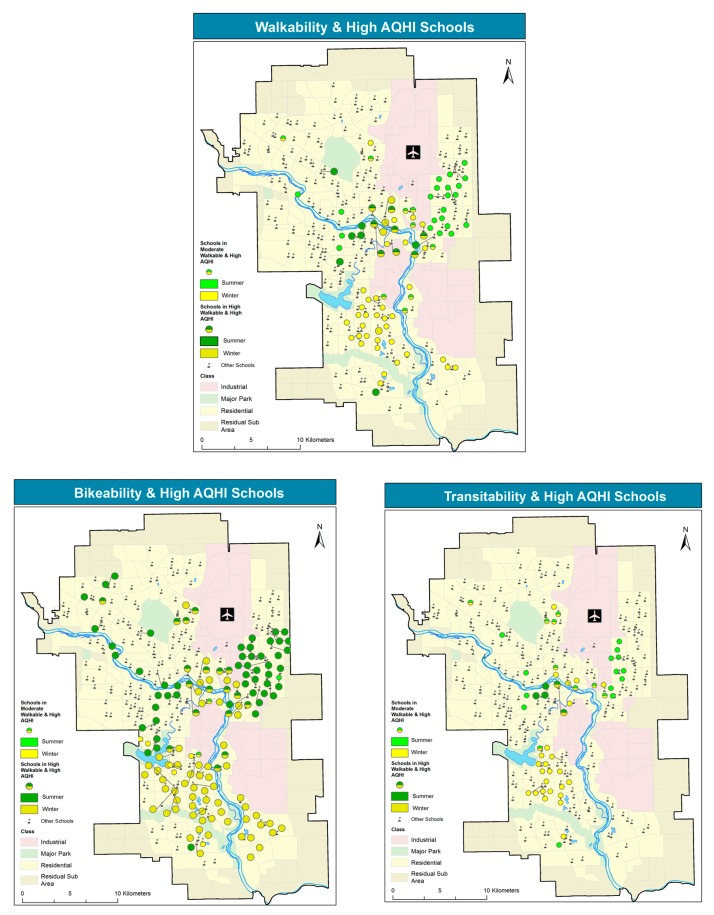
Schools in high pollution, walkable, bikable, and transitable locations.

The latter results, synthetically presented in Table 2, are mapped in Figure 6, providing greater spatial detail. This spatial analysis allows for identifying those schools that lie in locations with high air pollution and high active transportation scores.

All the maps point to a spatial dichotomy, as pollution levels are higher in the NE in the summer and in the south in the winter. Therefore, a larger portion of walkable schools lies in higher summer pollution locations in the NE, vs. a higher portion of walkable schools in winter pollution in the south. Walkable schools in and around the downtown core are exposed to relatively high pollution levels in both seasons. A very similar pattern is observed for transitability. Conversely, the bikability map exhibits a similar spatial pattern, yet, compared to the other active transportation modes, it features the largest portion of schools with high scores in high pollution locations, in both seasons.

## 4. Discussion

This exploratory analysis contributes to the literature on air pollution and public health at the intra-urban scale with a spatial thinking approach to school location with respect to air pollution and active transportation options [52,53]. The analysis indicates that Calgary’s air quality varies substantially across space, as do active transportation options. Consequently, intensity of walking and biking, along with air pollution exposure levels, vary for school children, according to the location of their school. Local and anecdotal evidence suggests that not only the general public, but also health practitioners, tend to be unaware of these spatial variations in exposure levels.

### 4.1. Air Pollution and School Location

Air pollution estimates for this study were obtained from LUR models. Despite the advantage of their high spatial resolution, these estimates are affected by some uncertainty. Acknowledging this limitation, we proposed a pseudo-air quality health index (p-AQHI), which yields a conservative estimate of air pollution. Nonetheless, p-AQHI yields values considered low- or moderate risk even by the standard AQHI. Within these safe levels, air pollution exhibits a distinct seasonal pattern, overall characterized by higher pollution in the winter, and by different spatial patterns in the two seasons. The study findings identify and present cartographically two layers of concerns. The first is with schools with poor active transportation and relatively poor air quality, located mostly in the northeast and in the south. This spatial pattern is likely associated with seasonal pollution sources and prevailing winds. As a consequence, schools in the south are exposed to higher air pollution in the winter, that is, during the school year, which should draw the attention of the board of education. Conversely, schools in the northeast, where air pollution is higher in the summer, pose lesser concerns, and more so to families and local communities. The second concern is with schools that enjoy good active transportation, yet poor air quality. These locations may pose even greater health risks, as they are conducive of greater physical activity in more polluted environments. The spatial analysis shows that most of these schools are located in and around the downtown core, which was developed before the 1950s, with high walkability and public transit options. As a hub of economic activities, an average of about 75,000 vehicles daily [54] travel between this core and the periphery. Hence, pollution in the area is relatively high in both seasons. Even though winter weather can be harsh in Calgary, posing health risks beyond air pollution exposure [55], there is no evidence of significant impact of the weather on physical activity [56,57].

### 4.2. Measuring Active Transportation

This exploratory study only considered active transportation modes in children’s commute to school. Other important forms of outdoor physical activity for children, such as playing or practicing outdoors sports, were not analyzed, and should be included in further analyses. Additionally, we only looked active modes of commuting to school, whereas children do walk and bike for recreation and other purposes.

Within these limitations, we had to choose measures of transitability [58,59,60]. Appropriate measures, e.g., PTAL (Public Transit Accessibility Levels), could not be calculated from the data available for this study. Walkscore™ is a useful, free-to-use, publicly available tool. There is debate around different measures, yet it is considered valid, reliable, and a reasonable proxy for walking behavior in multiple geographic locations and at multiple scales, with potential for public health research [24,25,35,40,61]. Walkscore is a robust and transferable measure, but it has its own limitations. Among others, it ignores topography, road connectivity, traffic, safety, and the impact of weather, in addition to air pollution [40]. More importantly, walkscore is not specific to children, their behaviour and destinations. For example, walkscore assigns low scores to large parks, which provide many opportunities for safe and healthy walking and biking, yet the sheer size of the park increases its distance from destinations considered by the index. Interestingly, however, the threshold distance of 1.6 km (1 mile) is used by walkscore to determine walkability, it has been used in studies of children’s mode of travel to and from school [62,63], and it is used by the Calgary Board of Education (CBE) as a benchmark to position bus stops for elementary school children [42]. Further research is needed to validate the more recently emerged bikescore and transitscore, which are available from the same source as walkscore [35]. Bikescore was recently used to analyze cycling behaviour [64]. In Calgary, the spatial variation of bikescore across the city suggests it may be a useful tool for planning bicycle infrastructure. Parts of the bikescore methodology was developed by a CIHR (Canadian Institute of Health Research)-funded project at Canadian Universities [35].

### 4.3. Schools, Car Dependence, and Air Pollution

The lack of active transportation options, or car dependence, is widespread and generally acknowledged as an existing and growing problem, as Calgary is sprawling, with a faster population growth in the suburbs than in the inner city, leading to more and more people driving over increasing distances. Further, the suburban population is largely formed by young families; indeed, children are the population segment most associated with car-dependence, as shown by the correlation analysis (Table 1). Often, children travel long distances to school, and, at times of shrinking resources, the school bus system is reducing their service [65], forcing families to drive children to the bus stop. With almost half of the schools (125 out of 279) in car-dependent locations, private vehicle and school bus traffic is high, posing potential health risks not only to children, but also to teachers, staff, and parents. The relationship between car-dependence and air pollution is intuitive, yet complex. Recent literature suggests a link between air pollution exposure and mode of active transportation [53,66]. The spatial analyses conducted in this study indicate that car-dependence and high air pollution tend to overlap spatially, particularly in the summer. This co-occurrence is less apparent in the winter, when other pollution sources, e.g., residential heating, and stronger winds confound air pollution patterns. The association between air pollution and transportation is also embedded in the LUR estimates, where traffic volumes are significant predictors. This study shows that the spatial pattern of car-dependence varies according to the various form of active transportation. More advanced spatial analytical techniques can model simultaneously the various forms of active transportation, as well as implement methods to extend the analysis from individual DAs to neighbouring units.

### 4.4. Recommandations and Future Work

The spatial knowledge emerging from this exploratory analysis can be effectively communicated, using maps, to the public, families, health practitioners, and school boards, to improve public health in school children. It is our intention to share these results with the Calgary Board of Education, the City of Calgary, and Alberta Health Services. These findings can help them understand *where* walking and biking is safer. Further, the study can help families implement more breathe-safe walking and biking, and assist in the development and enforcement of policies to improve respiratory outcomes in school-aged children. We offer some examples. LUR models have shown that some pollutants, particularly traffic-related particulate matter (PM_2.5_), decline rapidly as distance from roads increases: a simple precaution is to walk or bike on dedicated paths or minor roads, further from the main traffic flows. Avoiding walking small children near idling school buses can be achieved, for example, by crossing the street and walking on the other side. Without major infrastructure change, schools can redefine playgrounds and compounds, to encourage children to play far from idling buses and vehicles during the morning drop-off.

Further research is needed to improve knowledge of air quality and active transportation at school locations. While this study analyzes seasonal variations in air pollution, more work is needed to understand daily and hourly air pollution patterns at high spatial resolution, with air quality monitors placed in schoolyards or in their vicinity, to monitor pollution patterns at busy traffic times, such as children drop-off and pick-up. Further analysis shall consider the design and management of school bus routes, to take into consideration existing and potential active transportation options, as well as current and projected air pollution levels. Such work should also consider the redesign of school bus parking areas, to achieve greater separation between such areas and school playgrounds. The preliminary findings of this study are a starting point to analyze the impact of pollution in moderate to high walkable areas on respiratory health problems, child growth and cognitive skills. Based on the findings of this study, policy recommendations include promoting an increased use of public transit, enforcing idle bans, implement policies on zero emission by school buses and public transit, banning diesel buses, and ride-share policies. Schools enjoy perhaps the greatest potential to share these messages, by educating the young generations.

## 5. Conclusions

An exploratory analysis of school location with respect to air pollution and active transportation options was conducted for the city of Calgary, Canada. The study contributes to the literature on air pollution and public health at the intra-urban scale with a spatially explicit approach. The analysis shows that air quality exhibits spatial variation, resulting in varying levels of exposure to air pollution for different schools. The study findings include spatial analyses and maps that effectively communicate, in great spatial detail, varying levels of concern with school location, air pollution, and active transportation options. Concerns exist with schools that have poor air quality and poor active transportation: maps show that these schools are located primarily in the south, where air pollution is higher in the winter, i.e., during the school year; and in the northeast, where pollution is higher in the summer. Further concerns exist with schools that have good active transportation, yet poor air quality. The maps show that most of these schools are located in and around the downtown core, where air pollution is relatively high in both seasons. These results constitute novel knowledge, which, through the cartographic representation, can be effectively communicated to the public, health practitioners, and school boards. Further, the methods presented in this paper, developed for a safe air quality environment, can be applied for the analysis and management of more critical situations, and assist in the development of spatially oriented public health policies.

## Figures and Tables

**Table 1:** Correlation of air pollution, child and total population, and active transportation indices.

Variables	Pseudo-AQHI (Air Quality Health)		Population and Children			Active Transportation Indices		
	Summer AQHI	Winter AQHI	Total Pop	Age 0–14	Age 15–18	Walkability	Bikability	Transitability
S. AQHI	1.00	0.30	−0.18	−0.21	−0.20	0.19	0.06	0.20
(*p*-value)	0.00	0.00	0.02	0.01	0.01	0.02	0.85	0.01
W. AQHI	0.30	1.00	−0.19	−0.23	−0.20	0.08	−0.02	0.11
(*p*-value)	0.00	0.00	0.02	0.00	0.01	0.80	1.00	0.39
Total Pop	−0.18	−0.19	1.00	0.95	0.87	−0.26	−0.14	−0.27
(*p*-value)	0.00	0.00	0.00	0.00	0.00	0.00	0.12	0.00
Age 5–14	−0.21	−0.23	0.95	1.00	0.89	−0.36	−0.15	−0.36
(*p*-value)	0.00	0.00	0.00	0.00	0.00	0.00	0.08	0.00
Age 15–18	−0.20	−0.20	0.87	0.89	1.00	−0.38	−0.16	−0.32
(*p*-value)	0.00	0.20	0.00	0.00	0.00	0.00	0.06	0.00
Walk	0.19	0.08	−0.26	−0.36	−0.38	1.00	0.46	0.63
(*p*-value)	0.00	0.08	0.00	0.00	0.00	0.00	0.00	0.00
Bike	0.06	−0.02	−0.14	−0.15	−0.16	0.46	1.00	0.25
(*p*-value)	0.28	0.70	0.02	0.01	0.01	0.00	0.00	0.00
Transit	0.20	0.11	−0.27	−0.36	−0.32	0.63	0.25	1.00
(*p*-value)	0.00	0.08	0.00	0.00	0.00	0.00	0.00	0.00

**Table 2:** School location, air pollution, and active transportation modes.

	Walkability					Bikability					Transitability				
**Summer p-AQHI**		No	Low	High	Total		No	Low	High	Total		Some	Good	Exc.	Total
	Low	20%	18%	4%	42%	Low	0%	3%	38%	42%	Low	26%	15%	0%	42%
	Med.	16%	13%	5%	33%	Med.	0%	2%	31%	33%	Med.	19%	13%	1%	33%
	High	9%	**11%**	**5%**	25%	High	0%	**1%**	**24%**	25%	High	15%	**8%**	**2%**	25%
	Total	45%	41%	14%	100%	Total	0%	6%	93%	100%	Total	61%	37%	3%	100%
**Winter p-AQHI**		No	Low	High	Total		No	Low	High	Total		Some	Good	Exc.	Total
	Low	19%	15%	5%	39%	Low	0%	3%	36%	39%	Low	27%	13%	0%	39%
	Med.	14%	10%	5%	29%	Med.	0%	1%	28%	29%	Med.	15%	12%	2%	29%
	High	11%	**16%**	**4%**	32%	High	0%	**3%**	**29%**	32%	High	19%	**12%**	**1%**	32%
	Total	45%	41%	14%	100%	Total	0%	6%	93%	100%	Total	61%	37%	3%	100%

The % in bold shows high p-AQHI (summer or winter) where active transportation is either low or high.
